# Changes in shooting accuracy among basketball players under fatigue: a systematic review and meta-analysis

**DOI:** 10.3389/fphys.2025.1435810

**Published:** 2025-02-26

**Authors:** Shuairan Li, Yuanyuan Luo, Yingying Cao, Feng Li, Haodong Jin, Jing Mi

**Affiliations:** ^1^ Sports Coaching College, Beijing Sport University, Beijing, China; ^2^ Department of Physical Education, Dazhou College of Traditional Chinese Medicine, Dazhou, China; ^3^ School of Physical Education, National University of Malaysia, Kuala Lumpur, Malaysia; ^4^ School of Sports, Xi an University, Xi an, China; ^5^ China Basketball College, Beijing Sport University, Beijing, China

**Keywords:** basketball players, fatigue, shooting accuracy, systematic review, meta-analysis

## Abstract

**Objective:**

To investigate the influence of physical and mental fatigue of different intensities (mild, moderate or severe) on basketball shooting accuracy, with the aim of informing more effective training protocols and competition strategies.

**Methods:**

Literature searches were conducted on Web of Science, PubMed, and EBSCO databases up to 25 June 2024. Inclusion and exclusion criteria were specified, and data extraction sheets were prepared. Study quality was assessed by using the Cochrane Risk of Bias Tool in Review Manager 5.4, and Stata18.0 software was used for heterogeneity analysis, subgroup analysis, forest plots, stratification analysis, and bias assessment.

**Results:**

Moderate physical fatigue affected two-point shooting accuracy (*P < 0.01),*severe physical fatigue affected both two-point (*P = 0.02*) and three-point shooting accuracy (*p* < 0.01),with severe physical fatigue showing a greater detrimental impact on three-point shooting accuracy, while two-point shooting accuracy may vary under specific conditions. Additionally, adolescent athletes were less affected by severe physical fatigue compared to adult athletes or those with longer training experience. Moderate mental fatigue also significantly reduced free-throw accuracy (*p* < 0.01).

**Conclusion:**

The shooting accuracy of basketball players was significantly affected by moderate and severe physical fatigue. Severe physical fatigue notably adversely affected the accuracy of three-point shooting relative to moderate fatigue; Additionally, moderate mental fatigue significantly reduced free-throw accuracy, which may be attributed to a decline in cognitive executive functions, highlighting the importance of fatigue management in sports training.

**Systematic Review Registration:**

https://www.crd.york.ac.uk/prospero/#myprospero, identifier CRD42024539553

## 1 Introduction

Shooting is known as a critical and frequently used skill in basketball, which directly influences the outcome of a game. Player scoring primarily relies on jump shots, layups, and free throws, with mid- to long-range jump shots and free throws accounting for a significant proportion of points (Wang and Zheng, 2022). These are the primary scoring methods and have a crucial impact on the game results.

Basketball is a high-intensity intermittent team sport characterized by frequent sprinting, sliding, and jumping (Qarouach et al., 2024; Stojanović et al., 2018). During high-intensity basketball games, athletes exercise at high intensities for approximately 15% of the game time with an average heart rate of 169 ± 9 beats per minute, nearing 90% of the maximum heart rate (García et al., 2020; Puente et al., 2017; Scanlan et al., 2011; Vázquez-Guerrero et al., 2019). The heart rate of athletes remains above 85% of the maximum in about 75% of the game time; energy is predominantly supplied by glycolysis, and blood lactate concentration is 6–7 mmol/L, indicating a linear increase in heart rate and blood lactate concentrations during intense games (Mcinnes et al., 1995; Vencúrik, 2016). Consequently, basketball players inevitably experience physical (Pernigoni et al., 2024a) and mental fatigue (Cao et al., 2021), and they usually need to shoot under such situation. Maintaining a high-level shooting performance during intense games is crucial for victory.

The impact of fatigue on basketball shooting accuracy has been extensively studied, and it is commonly believed that shooting accuracy is associated with changes in shooting technique caused by fatigue. Erčulj and Supej (2009) reported that moderate-to-high fatigue may lead to alterations in arm and shoulder biomechanics. However, as indicated by Uygur et al. (2010), there are minimal changes in biomechanical parameters under progressively increasing physiological loads. Li et al. (2021) have observed that female basketball players exhibit increased angular velocities in lower limb joints and decreased upper limb velocities under fatigue conditions. Notably, the angular velocities of the right wrist and elbow joints significantly decrease post-fatigue, which is considered a critical factor of the reduced final shooting accuracy. Additionally, mental fatigue can also affect shooting technique. Accumulating studies have shown that mental fatigue can lead to reduced concentration, judgment, and reaction speed and thus cause unstable shooting movement, thereby affecting shooting accuracy (Alarcón et al., 2017; Faro et al., 2023; Metulini and Le Carre, 2020).

Most existing studies have focused on the impact of physical fatigue on basketball shooting skills, and less attention is paid to the role of mental fatigue. Moreover, the effects of fatigue of varying levels and types on shooting performance remain underexplored. Hence, our research investigated the effects of different forms and intensities of fatigue on shooting accuracy in basketball players, aiming to provide coaches and athletes with actionable and data-driven insights into adjusting training and gameplay strategies, ultimately enhancing shooting accuracy, training efficiency, and overall performance.

## 2 Methods

This study conforms to all PRISMA guidelines (Page et al., 2021) and reports the required information accordingly (see Supplementary Checklist, http://links.lww.com/PHM/C247). This research program has been registered on the PROSPERO System Evaluation Registration Platform, registration number: CRD42024539553 (05/05/2024).

### 2.1 Literature search strategy and screening process

Literature searching was conducted on the Web of Science, PubMed, and EBSCO databases, covering all records from the inception of each database to 25 June 2024. This scoping review was conducted following the PRISMA guidelines (Page et al., 2021) Key terms and their synonyms were systematically identified using the MeSH database by authors with expertise in the investigated area. These key terms include, but are not limited to “fatigue”, “physical fatigue”, “mental fatigue”, “localized fatigue”, “fatigue level”, “degree of fatigue”,“shooting accuracy”, “shooting hit rate”, “shooting”, “performance”, “shooting percentage”, “shooting efficiency”, “field goal percentage”, “basketball player”, and “basketball athlete”. As reported in Supplementary File S1 (“Search strategy”),these terms were used in various combinations across the databases, utilizing Boolean search operators (AND, OR).

### 2.2 Selection criteria

The study’s inclusion criteria were further defined. The PICOS framework was used to identify the core elements of the research. The population (P) consisted of professional male basketball players from national or international leagues. The intervention (I) involved the scientifically validated methods used to induce either physical fatigue (such as prolonged physical training) or mental fatigue (such as high-intensity cognitive tasks), with methods that combine both physical and mental interventions excluded. In the control group (C),the condition corresponding to the experimental group’s fatigued state is defined as the non-fatigue control condition [in randomized controlled trails (RCTs)]or the non-fatigued,baseline state (in pre-post experimental designs). The outcome (O) focused on the shooting accuracy, primarily assessing changes in shooting performance using detailed statistical analyses. The study design (S) was limited to randomized controlled trials and prospective cohort studies to ensure the reliability of causal relationships, and cross-sectional studies were excluded.

The specific inclusion criteria were: (1) the study population consisting of basketball players in a healthy condition; (2)interventions that involved inducing either physical or mental fatigue; (3) studies utilizing the ratio of goals scored to total attempts for outcome measures, with the primary outcome being the shooting accuracy; (4) Randomized Controlled Trials and Pre-Post experimental designs. (5) study reports published in English. (6) Based on general guidelines regarding the use of RPE in sports (Eston, 2012), we decided to categorize fatigue levels as follows: “mild”, when RPE ≤ 12 (CR-20), or RPE ≤ 4 (CR-10). “Moderate”, when RPE = 13–16 (CR-20) or RPE = 5–6 (CR-10). “Severe”, when RPE ≥ 17 (CR-20) or RPE ≥ 7 (CR-10). Additionally, physiological parameters were also considered when categorizing fatigue levels, as described in Table 1. The exclusion criteria were: (1) participant including wheelchair basketball player or members of injury rehabilitation and other special groups; (2) interventions including elements other than physical or mental fatigue, such as strength training, nutritional supplements, and pharmacological treatment; (3) studies utilizing basketball robots or artificial intelligence for basketball game measures; (4) studies with incomplete data for analysis; (5) qualitative research, case reports, review articles, non-intervention studies, and conference papers; (6) interventions combining the induction of both physical and mental fatigue [e.g.,physical-mental dual fatigue induction, where physical and mental loads are applied simultaneously or sequentially (Chen et al., 2023)]. ”.

**TABLE 1:** Basic characteristics of the included studies.

Included studies	Nation	Sample characteristics	Study design	Fatigue induction	Shooting distance	Scoring method	Outcome measure (%)
Alarcón et al. (2017)	Spanish	Male,n = 18,、Age/21 ± 2.5 years TE/10.2 years	Double arm	Moderate mental fatigue in participants was induced using the N-back task. Fatigue Definition Methods and Tools: ① A score between 40 and 60 on the NASA TLX scale; ② A decline in accuracy by 5%–15% and a delay in reaction time of approximately 10%–30% on the 2-back task are considered indicative of moderate mental fatigue.	FT	G/T Ratio	FG
Ardigò et al. (2018)	Italy	Male,n = 24,Age/16 et a years TE/9 ± 2.6 years	Single arm	Moderate/heavy physical fatigue through round-trip running intensity, Yo-Yo running 560 and 1600 m Fatigue Definition Methods and Tools: Gradually increasing from 50% to over 85% of maximum heart rate is considered indicative of moderate to severe physical fatigue.	3 PS	G/T Ratio	FG
Aydemir and Cinar (2019)	turkey	Male,n = 10,Age/16 ± 0.5 years	Single arm	Moderate to severe physical fatigue was achieved through shuttle run intensity, with 20 m acceleration sections and 5 m active recovery phases in the Yo-Yo test.	2 PS 3 PS	G/T Ratio	FG
Bahrami et al. (2020)	Iranian	Male,n = 18,Age/22 ± 3.4 years TE/3 years	Single arm	Moderate mental fatigue was induced in participants through the Stroop task and mathematical calculations. Fatigue Definition Methods and Tools: A VAS score ranging from 4 to 6 is considered indicative of mental fatigue.	3 PS	G/T Ratio	FG
Brini et al. (2021)	Italy	Male,n = 16,Age/23 ± 2.8 years TE/11 ± 3.9 years Player Positions: The number of players in each position is equal.	Single arm	Moderate physical fatigue was induced through ten 30-m shuttle sprints with recovery training. Fatigue definition and measurement tools: ① Continuous heart rate monitoring (HR = 135 ± 10 bpm); ②Rating of Perceived Exertion (RPE = 14 ± 2)	3 PS	G/T Ratio	FG
Bourdas et al. (2024)	Greece	Male,n = 38,Age/24 ± 2.7 years TE/12 ± 2.7 years Player Positions Include: Guards, Forwards, and Centers	Single arm	Severe physical fatigue was induced through training activities including standing, walking, running, and sprinting. Fatigue Definition Methods and Tools: ① Perceived Exertion (RPE ≥ 18); ② Respiratory Exchange Ratio (RER ≥ 1.1), ③ Heart Rate ≥ 90% HRmax	3 PS	G/T Ratio	FG
Cengizel, et al. (2023)	Italy	Male,n _**U12**_ = 35、Age/11 ± 0.6 years; n _**U14**_ = 34、Age/12 ± 0.5 years; n _**U16**_ = 20、Age/14 ± 0.4 years; n _U18_ = 10, Age/16. ±0.3 years	Single arm	Moderate physical fatigue was induced using a 20-m shuttle run with incremental loads. Fatigue definition and measurement tools: The Rating of Perceived Exertion (RPE) is used, with a value of 15 ± 1 indicating the onset of moderate fatigue.	FT 3 PS	G/T Ratio	FG
Englert et al. (2015)	Germany	Male,n = 38,Age/29 ± 4.9 years	Double arm	Moderate mental fatigue was induced in participants by having them transcribe texts while consistently omitting the most common letters in German, “e” and “n,” to challenge their writing habits. Fatigue Definition Methods and Tools: ① Control Checklist Scale ② Emotional Scale.	FT	G/T Ratio	FG
Filipas et al. (2021)	Italy	Male,n = 19,Age/20 ± 3.0 years	Single arm	Moderate mental fatigue was induced by having participants watch a 30-minute basketball tactics video until they fully understood the strategies and techniques, followed by answering 12 mediums to complex questions related to the video. Fatigue Definition Methods and Tools: A score between 4 and 6 on the Visual Analogue Scale (VAS) is used to define moderate mental fatigue.	FT	G/T Ratio	FG
Marcolin et al. (2018)	Italy	Male,n_(AdultGroup)_ = 11,Age/26 ± 6 years TE ≥ 10 years n_(YouthGroup)_ = 10, Age/18 ± 1.0 years,TE ≥ 5 years Player Positions Include: guards, forwards, and centers.	Single arm	Moderate to severe physical fatigue was induced through training exercises including running, vertical jumps, shooting, and sprinting. Fatigue Definition Method and Tools: ① Heart Rate: Above 95% of maximum heart rate or between 85% and 95%; ② Blood Lactate Concentration: Between 5.75 ± 1.25 and 6.22 ± 1.34 mmol L^-1^, which is considered indicative of severe to extreme physical fatigue.	2 PS 3 PS	G/T Ratio	FG
Pojskic et al. (2018)	Sweden	Male,n = 38.Age/19 ± 2.9 years TE/7 ± 2.6 years Player Positions Include: All perimeter players.	Single arm	Severe physical fatigue was induced through 17 min of general warm-up and basketball shooting drills. Fatigue definition and measurement tools: The total sprint time refers to the cumulative time of six sprints. If the Fatigue Index (FI) reaches or exceeds 25%, it is considered indicative of severe physical fatigue.	FT 2 PS 3 PS	G/T Ratio	FG
Padulo et al. (2018)	Italy	Male,n = 22,Age/16 ± 0.9 years TE/8 ± 3.0 years	Single arm	Moderate/heavy physical fatigue through round-trip running intensity, Yo-Yo running 540 and 1620 m Fatigue Definition Methods and Tools: Gradually increasing from 50% to over 85% of maximum heart rate is considered indicative of moderate to severe physical fatigue.	2 PS	G/T Ratio	FG
Slawinski et al. (2018)	French	Male,n = 8,Age/16 ± 1.2 years TE/11.8 ± 3.9 years	Single arm	Severe physical fatigue was induced by a 20-m run with acceleration/deceleration, followed by five consecu tive maximal vertical jumps. Fatigue definition and measurement tools: A heart rate reaching 85% of the maximum heart rate is defined as the onset of severe physical fatigue.	3 PS	G/T Ratio	FG
Shaabani et al. (2020)	America	Male,n = 18,Age/28 ± 4.5 years TE/7 ± 2.3 years	Double arm	Moderate mental fatigue in participants was induced via the Stroop task and mathematical calculations. Fatigue Definition Methods and Tools: ; Depletion Sensitivity Scale (DSS): A score of 3 indicates moderate mental fatigue	FT	G/T Ratio	FG

Note:FG, field goal; G/T, goals to total ratio; FT, free throw,2 PS, two-point shots; 3 PS, three-point shots; TE, training experience.

### 2.3 Data extraction and quality assessment

In this meta-analysis, data were screened and extracted by two researchers independently; a predefined table was used to systematically record and encode the information. The extracted details included: (1) basic literature information such as authors, nationality, and publication year; (2) participant details including sample size, age, gender, years of training duration, competition experience, and level of performance; (3) intervention measures, encompassing study design, fatigue assessment methods, fatigue types, induction methods, and fatigue levels; (4) outcome measures, specifically the ratio of shots-made and shooting accuracy (mean ± SD); (5) studies that involved multiple shooting distances were considered to contain multiple fatigue interventions; (6) fatigue categorization, encompassing mild, moderate, and severe levels; (7) fatigue intervention scheduling including warm-up, relaxation activities, and main training periods. The literature quality and potential publication bias were assessed using the Cochrane Collaboration’s Risk of Bias Tool (Liew et al., 2020), covering several aspects as follows: (1) random sequence generation; (2) allocation concealment; (3) blinding of participants and personnel; (4) blinding of outcome assessment; (5) completeness of outcome data; (6) selective reporting of results; and (7) other sources of bias. If a study was assessed as “low risk” across all domains, it was considered to have a low overall risk of bias. If one to two domains in a study were judged as “high risk”, or “unclear risk,” the study was considered to have a moderate overall risk of bias. Studies with more than two domains judged as “high risk” or “unclear risk” were considered to have a high overall risk of bias. These evaluations were performed independently by researchers Li and Luo, and any disagreements were resolved through discussion or by consulting a third researcher, Cao.

### 2.4 Statistical methods

Statistical analyses (including pooling effect sizes, subgroup analysis, sensitivity analysis, and regression analysis) were conducted using Stata18.0 software. Outcome measures were calculated as mean ± SD and the 95% confidence intervals (CI) were estimated. Statistical significance was determined at *p* < 0.05 and *p* < 0.01 (Borenstein et al., 2021). Moreover, the Q test and I^2^ test were performed to assess heterogeneity among the included studies. Homogeneity was assumed when the p-value from the Q test was > 0.1 and I^2^ < 50%, and a fixed-effect model was used (Cheung and Cheung, 2016). When significant heterogeneity was observed, a random-effects model was employed (Zhang et al., 2019). Hedges’ g effect sizes were calculated, and classified as small (0.2–0.5), medium (0.5–0.8), or large (>0.8) (Hedges and Tipton, 2010). For small samples, the correction factor formula proposed by Hedges and Tipton, 2010 was applied to reduce estimation bias. In the presence of significant heterogeneity, further analyses (such as subgroup analysis, sensitivity analysis, and meta-regression) were conducted. Publication bias was explored through funnel plots and Begg and Egger’s tests (Hedges and Tipton, 2010). For studies numbering fewer than 10, the trim-and-fill method was utilized to adjust potential publication bias (Sera et al., 2019).

## 3 Results

### 3.1 Literature search results

**FIGURE 1:**
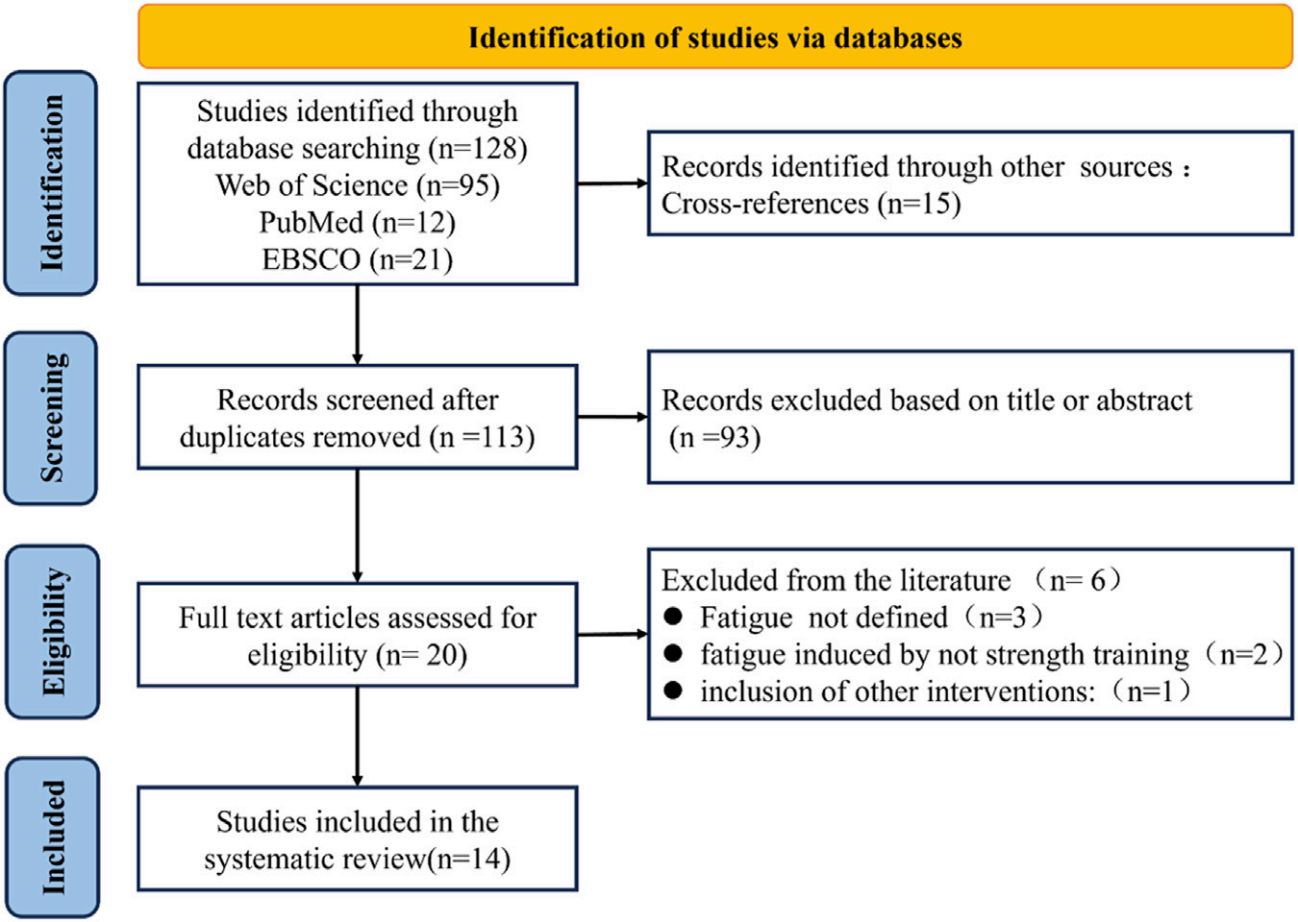
PRISMA flow diagram.

After a comprehensive search across the Web of Science, PubMed, and EBSCO databases, a total of 128 articles were identified. An additional eight articles were screened through manual searching. Next, these records were imported into EndNote X7, and the duplicates were removed. After that, 113 articles remained. A preliminary screening based on titles and abstracts resulted in 20 articles. Subsequently, following further screening through a full-text review, 14 quantitative studies were finally included in this study for analysis (Figure 1).

### 3.2 Study characteristics and quality assessment

**FIGURE 2:**
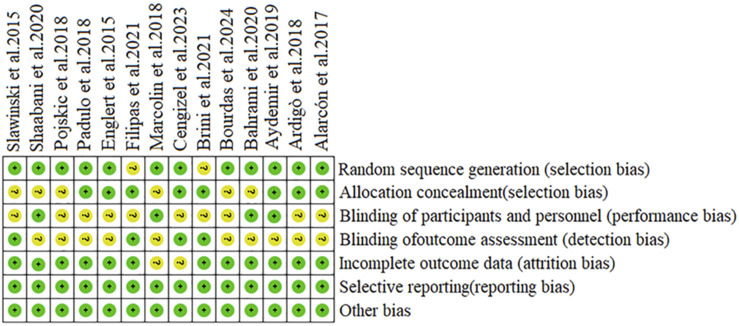
Quality assessment results of the included studies.

A total of 14 studies (3 double-arm and 11 single-arm studies) were incorporated in this meta-analysis, which pertained to physical fatigue (n = 9) and mental fatigue (n = 5). Given that some studies comprised multiple independent experiments, each experiment was treated as a separate research entity. In total, 388 participants (173 adolescents and 215 adults) were involved. Methods to induce physical fatigue included sprint shuttle runs, standing long jumps, and shooting drills, while methods to induce mental fatigue involved novel writing tasks, basketball tactical video analysis, and cognitive fatigue tasks. In all studies, the shooting accuracy was assessed based on the ratio of successful shots to total attempts (Table 1). Regarding quality assessment (and based on the criteria outlined in section 2.3), 11 studies were considered to have a moderate risk of bias, and three studies were considered to have a high risk of bias. The specific assessment results are presented in Figure 2.

### 3.3 Meta-analysis of changes in shooting accuracy under fatigue conditions in basketball players

Our screening and selection process identified studies that induced moderate and severe physical fatigue. However, no studies addressing mild physical fatigue were found. Regarding psychological fatigue, only interventions inducing moderate levels were eligible for inclusion in our meta-analysis. As a result, the findings presented below pertain exclusively to moderate and severe physical fatigue, and moderate psychological fatigue.

#### 3.3.1 Meta-analysis of changes in shooting accuracy under moderate physical fatigue

**FIGURE 3:**
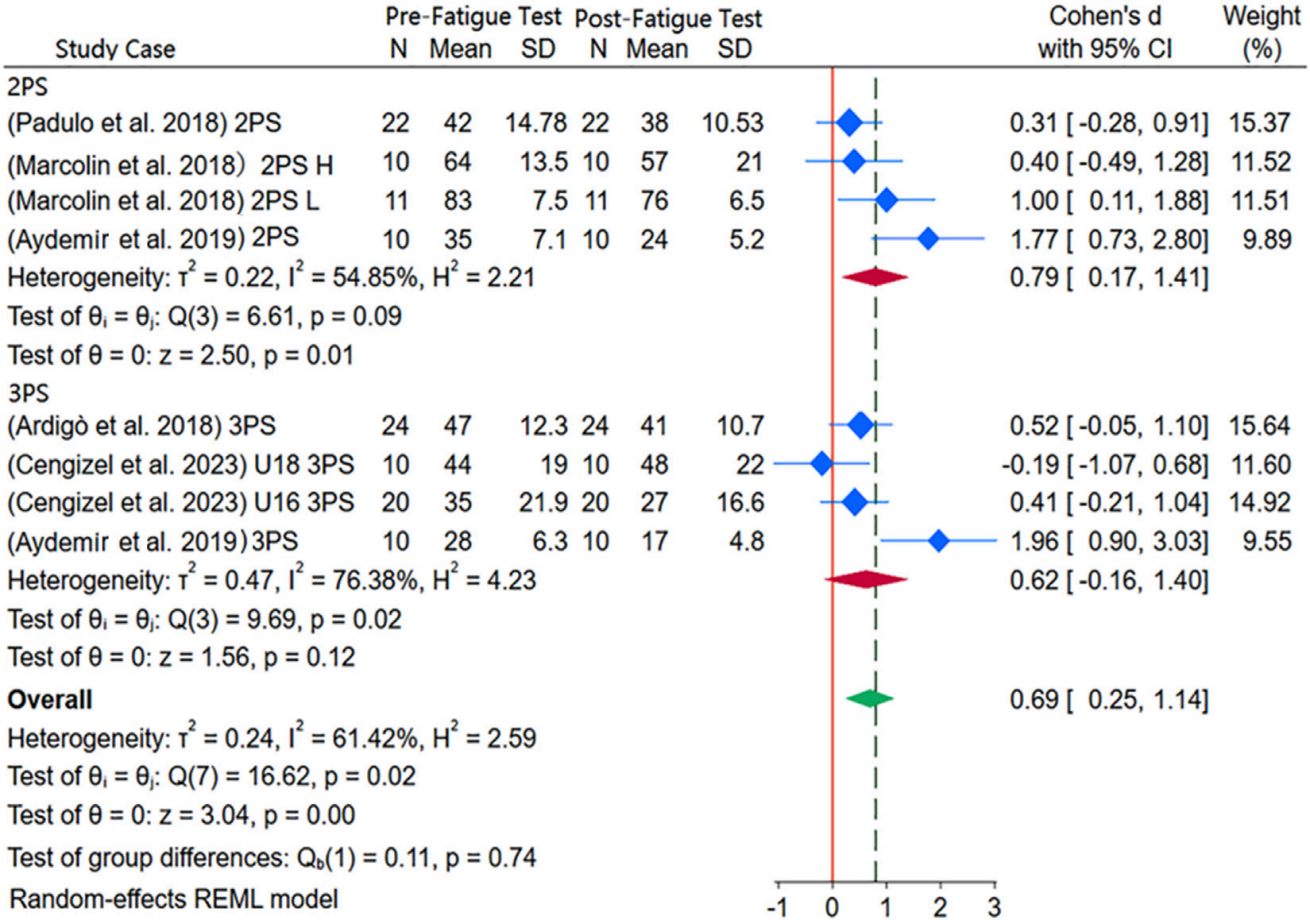
Subgroup meta-analysis forest plot of the impact of moderate physical fatigue on shooting accuracy.

As shown in Figure 3, a total of five papers, including eight independent studies, were included for studying the effect of moderate physical fatigue on shooting accuracy. There were two single-arm studies and one double-arm study about two-point shots, involving the high-level (H) and low-level (L) groups with a total of 53 participants. For three-point shots, there were two single-arm studies and one double-arm study, involving the U18 group (average age 16.0 ± 0.0 years) and U16 group (average age 14.20 ± 0.4 years), with a total of 64 participants. Shooting distance was used as a subgroup variable, revealing substantial heterogeneity (I^2^ = 60.66%), and thus a random-effects model was chosen for meta-analysis. Overall, moderate physical fatigue significantly reduced shooting accuracy (SMD = 0.67, 95% CI [0.24, 1.10], *p* < 0.01). In individual observations for two-point shots, the players had significantly higher pre-test scores than post-test scores under moderate fatigue (SMD = 0.76, 95% CI [0.17, 1.35], *p* = 0.01). However, for three-point shots, no significant difference was observed between the pre-test and post-test scores under moderate physical fatigue (SMD = 0.67, 95% CI [0.24, 1.10], *p* = 0.11). Additionally, there was no significant difference in shooting accuracy between two-point and three-point shots (*p* = 0.74). Although the number of studies was fewer than ten and the use of Begg and Egger tests was prevented, the trim-and-fill test confirmed no need for adjustments, ensuring data stability.

#### 3.3.2 Meta-analysis of changes in shooting accuracy under severe physical fatigue

**FIGURE 4:**
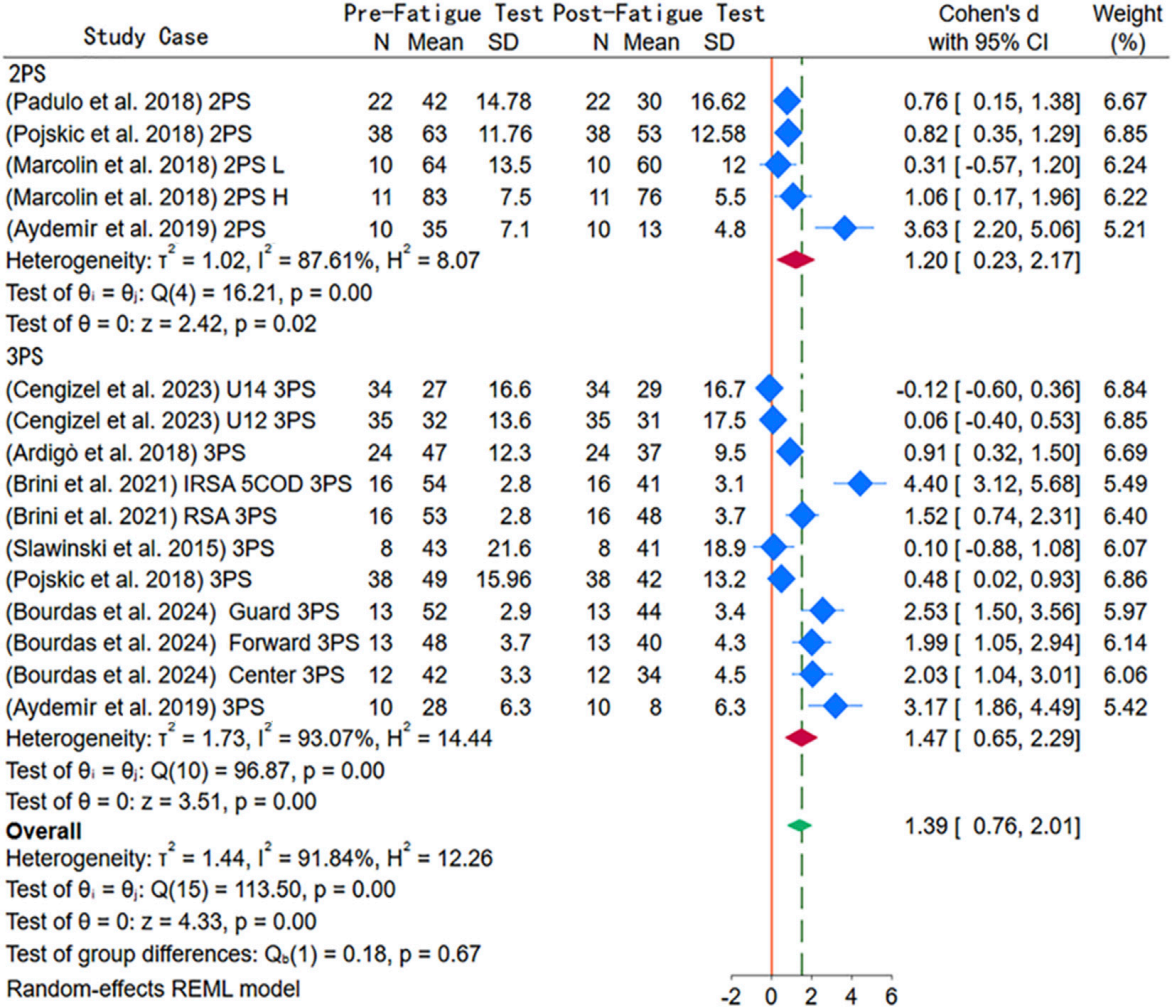
Subgroup meta-analysis forest plot of the impact of severe physical fatigue on shooting accuracy.

As indicated by Figure 4, nine articles were included for examining the impact of severe physical fatigue on shooting accuracy, including 14 independent studies. For two-point shots, there were three single-arm studies and one double-arm study, involving the high (H) and low (L) level groups, with a total of 91 participants. For three-point shots, there were four single-arm studies, two double-arm studies, and one triple-arm study, covering 505Change-of-Direction and Integrated Reactive Strength and Agility training, U18 and U16 age groups, and different positions (forward, center, and guard), with a total of 219 participants. Shooting distance was used as a subgroup variable, and significant heterogeneity (I^2^ = 91.84%) was observed, thus meta-analysis was performed using a random-effects model. Overall, severe physical fatigue significantly reduced the accuracy of both two-point and three-point shots (SMD = 1.39, 95% CI [0.76, 2.01], *p* < 0.01). Specifically, the pre-test accuracy of two-point shots was significantly higher than post-test (SMD = 1.20, 95% CI [0.23, 2.17], *p* = 0.02); moreover, the pre-test accuracy of three-point shots was also remarkably higher than post-test (SMD = 1.47, 95% CI [0.65, 2.29], *p* < 0.01). Additionally, no significant difference was found in the accuracy between two-point and three-point shots (*p* = 0.67). According to publication bias test results, both Begg’s test (Z = 3.38, *p* < 0.01) and Egger’s test (Z = 5.621, *p* < 0.01) indicated significant bias, but the trim-and-fill method found no studies requiring adjustment, confirming the data stability.

Through analyzing how age and years of training influenced the difference in shooting accuracy for two-point and three-point shots under severe physical fatigue, we further explored the sources of heterogeneity (Table 2). For two-point shots (SMD = 1.49, CI [-0.46, 3.43]), there was a trend towards reduced two-point shooting accuracy, but it was not statistically significant (*p* = 0.13). In contrast, data from the adult group showed that severe physical fatigue significantly reduced two-point shooting accuracy (SMD = 0.87, CI [0.46, 1.29], *p* < 0.01). In the analysis of training years, whether ≤8 years or >8 years, severe physical fatigue significantly reduced the two-point shooting accuracy. Regarding the analysis of three-point shooting accuracy, it was found that severe physical fatigue negatively impacted three-point shooting accuracy in both the youth and adult groups, with the adult group showing a more pronounced effect (SMD = 2.08, CI [1.07, 3.09], *p* < 0.01). Similarly, the analysis of training years showed that athletes with more than 8 years of training experienced a greater negative impact on three-point shooting accuracy (SMD = 2.06, CI [0.98, 3.13], *P* < 0.01).

**TABLE 2:** Stratified analysis of the impact of severe fatigue on shooting accuracy.

Research variables	Grouping variables	Number of studies/Entries	Heterogeneity test results			Meta-analysis results		Between-group differences
	Age/Training years		Q	P	I^2^/%	SMD(95% CI)	P	P
2 PS	Adolescent	3/3	15.9	0.00	92.6	1.49 [-0.46, 3.43]	0.13	0.55
	Adult	2/2	0.22	0.64	00.0	0.87 [0.46, 1.29]	0.00	
	≤8 years	2/2	0.99	0.32	0.00	0.71 [0.30, 1.12]	0.00	0.65
	>8 years	2/2	0.30	0.59	0.00	0.86 [0.35, 1.36]	0.00	
3 PS	Adolescent	4/5	26.45	0.00	92.54	0.73 [-0.34, 1.80]	0.18	0.07
	Adult	3/6	44.58	0.00	88.45	2.08 [1.07, 3.09]	0.00	
	≤8 years	2/2	1.28	0.26	21.71	0.65 [0.24, 1.06]	0.00	0.02
	>8 years	3/6	30.0	0.00	86.13	2.06 [0.98, 3.13]	0.00	

Note: ,2 PS, two-point shots; 3PS, three-point shots,; SMD: standardized mean difference; CI: confidence interval.

#### 3.3.3 Meta-analysis of changes in shooting accuracy under moderate mental fatigue

**FIGURE 5:**
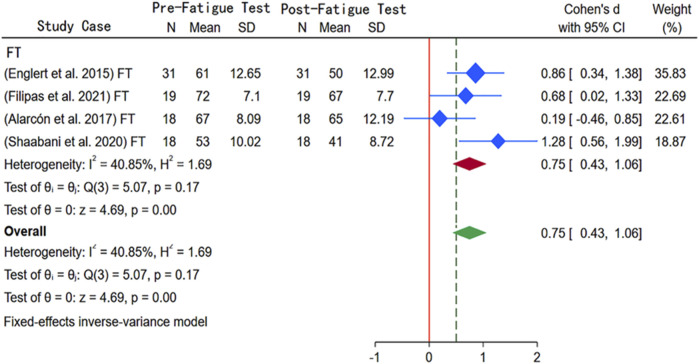
Subgroup meta-analysis forest plot of the impact of moderate mental fatigue on shooting accuracy.

In Figures 4, 5 studies examined the impact of moderate mental fatigue on shooting accuracy, involving 86 participants. With shooting distance as a subgroup variable, heterogeneity was calculated at 41.11%, and the Q-test p-value was approximately 0.18 (*p* > 0.1), so a fixed-effect model was used in the meta-analysis. The results indicated that moderate mental fatigue significantly decreased free-throw accuracy (SMD = 1.20, 95% CI [0.23, 2.17], *p* < 0.01). Despite some heterogeneity (I^2^ = 41.11%), no statistically significant difference was observed (*p* = 0.17). Additionally, as the included study numbers were fewer than 10, Begg’s and Egger’s tests were not conducted. However, the trim-and-fill test detected no studies requiring adjustment, suggesting that data robustness is reliable.

## 4 Discussion

This review investigated the negative statistically significant relationship between shooting accuracy and both moderate and severe physical fatigue or moderate mental fatigue. Specifically, under moderate fatigue, two-point shooting accuracy declines significantly, whereas three-point shooting accuracy remains unaffected. Severe physical fatigue adversely affected both three-point and two-point shooting accuracy. Furthermore, moderate mental fatigue contributed to a significant reduction in free-throw accuracy, which underscored the influence of mental fatigue on shooting performance.

### 4.1 The impact of physical fatigue on shooting accuracy

Ben Abdelkrim et al. (2010) have revealed that basketball players typically spend 8.8%, 5.3%, and 2.1% of their game time on high-intensity movements, sprinting, and jumping during the games. These high-intensity activities would negatively affect athletes’ heart rate, blood lactate (Stojanović et al., 2018), perceptual components of fatigue and performance (e.g., vertical jumping, sprinting) (Pernigoni et al., 2024a). Therefore, players are often required to perform shooting actions in a fatigued state, which may affect their shooting skills and accuracy (Matthew and Delextrat, 2009). Accumulating studies have demonstrated that physiological load indicators such as heart rate, blood lactate, blood/salivary cortisol, inflammatory markers and perceived exertion/fatigue can be extensively used to evaluate athletes’ fatigue levels during training and competition (Brini et al., 2021; Erčulj and Supej, 2006; Li et al., 2021; Pernigoni et al., 2024a; Erčulj and Supej, 2009). Consistently, Coutts et al. (2007) have suggested that delayed heart rate recovery and increased blood lactate concentration are biomarkers of physiological fatigue. Additionally, Micklewright et al. (2017) have also validated the effectiveness of the RPE scale in assessing athlete fatigue. Based on the above research on the reasonable definition of fatigue-related indicators, a graded evaluation of the fatigue intervention intensity and fatigue level was conducted on the included basketball players in this study.

Ardigò et al. (2018) have investigated a significant association between exercise intensity and shooting accuracy. Under severe fatigue conditions,it appears that high heart rate values (i.e., nearing maximal exertion) can significantly reduce shooting accuracy (p < 0.01). However, at a low heart rate (HRmax = 50%), no significant change in shooting accuracy is observed (*p* = 0.255). It has been shown that at lower heart rates, increased muscle temperature facilitates rapid contraction and relaxation of agonist and antagonist muscles, thereby enhancing muscle power output and response time, which benefits shooting accuracy (Padulo et al., 2018). However, this study revealed a significant reduction in shooting accuracy under moderate fatigue, and two-point shooting accuracy was more adversely affected than three-point shooting accuracy. This discrepancy may be attributed to the variations in experimental protocols. For instance, Marcolin et al. (2018) have pointed out that both master and rookie players under moderate fatigue experience a 7% reduction in two-point shooting accuracy, which recommends integrating high-intensity technical training into the training program to enhance shooting accuracy during competition.

According to an in-depth analysis of how severe fatigue affects shooting movement, fatigue primarily causes deformations in shooting movement, ultimately affecting shooting accuracy. Li et al. (2021) have indicated that the change of kinematic parameters under fatigue may lead to unstable shooting. Therefore, the reduction in maximal strength and power output is characterized by decreased upper limb angular velocity and increased lower limb angular velocity. The reduction in maximal strength and power output may be critical factors in the deformation of shooting actions. Enoka and Duchateau (2008) have revealed the effects of muscle fatigue on muscle function, as well as the causes and mechanisms of muscle fatigue. They report that muscle fatigue could be explained by various mechanisms, and different tasks may lead to different fatigue mechanisms. The primary mechanism suggests that muscle fatigue typically develops as a result of a reduction in maximal strength or power capabilities, suggesting that sub-maximal contractions can still occur after muscle fatigue sets in (Miller and Bartlett, 1993). This confirms that the deformation of shooting actions can be influenced by muscle fatigue. As shooting distance increases, the shooting angle may become smaller due to insufficient strength. Concurrently, shooting would require greater propulsive force to reach the basket, with an increased corresponding shooting speed (Caseiro et al., 2023; Elliott and White, 1989; Miller and Bartlett, 1996). Therefore, the reduction in joint angular velocity caused by upper limb fatigue during the motion undoubtedly has a great impact on long-distance shooting. Rupčić et al. (2020) have examined the effects of progressively increased physiological loads on joint angular velocities and shooting accuracy during basketball jump shots. The study primarily focused on fatigue-caused changes in lower and upper limb joint angular velocities, and the relationship between these parameters and the shooting duration and accuracy. This review reported the changing differences in the limb’s angular velocities and ball release height under increasing fatigue. However, no significant difference was observed in the kinematic parameters affecting the shooting duration and angle (Slawinski et al., 2018), but there was a notable decrease in shooting accuracy (Erčulj and Supej, 2006). Fatigue-induced reductions in shooting accuracy are linked to the decrease in shooting height and wrist joint angular velocity (Li et al., 2024; Li et al., 2021). Furthermore, elbow extension is crucial to be the most important part of the ball release phase and suggest that elbow extension is the determining contributor to ball velocity at release (Miller and Bartlett, 1993).

Existing literature has indicated the complex impact of age on fatigue. It has been shown that the adaptability of adolescents to training stimuli may vary due to the differences in their growth hormone levels, muscle and bone maturity (Gäbler et al., 2018). Adolescent athletes have a similar endurance level to adults; from a technical perspective, the shooting movements of adolescent basketball players gradually stabilize with age, and there are significant differences in shooting accuracy among different age groups under fatigue (Cengizel et al., 2023). From the perspective of muscle fiber composition analysis, children and adolescents have a lower proportion of type II muscle fibers and a higher proportion of type I muscle fibers, which makes them more resistant to fatigue (Oertel, 1988). They primarily rely on aerobic metabolism rather than anaerobic metabolism during the exercise. Nevertheless, this study revealed that in repeated-sprint training, the concentrations of anaerobic metabolic byproducts (such as hydrogen ions and phosphates) produced by children are lower than adults. High concentration and slower clearance rate of these metabolic byproducts make adults more prone to peripheral fatigue, with a longer fatigue duration (Allen et al., 2008). Additionally, In terms of metabolism, children and adolescents have a faster rate of phosphocreatine synthesis, stronger mitochondrial oxidative capacity, and a higher rate of ATP regeneration after high-intensity exercise relative to adults (Armstrong, 2018). This study suggests that the difference in shooting accuracy between adolescent and adult athletes under high-intensity physical fatigue may be associated with age. Similarly, athletes with more than 8 years of training experience are more vulnerable to the effects of severe fatigue, which has a greater impact on three-point shooting than on two-point shooting. The cumulative physical and psychological fatigue from years of competition experience likely contributes to this phenomenon. As athletes age and accumulate more years of training, they may require extended recovery periods to maintain optimal performance levels (Ben Abdelkrim et al., 2007).

Furthermore, when considering gender as a variable, it is evident that research on the shooting performance of female basketball players under fatigue is limited. To date, only one study has focused on elite female basketball players (Li et al., 2021). The study found that the mid-range jump shot accuracy decreased from 54.1% to 53.3% under fatigue, although the difference was not statistically significant. This suggests that shooting performance is influenced by both shooting distance and skill level. Elite female athletes may counteract the effects of fatigue by making technical adjustments, thus maintaining shooting efficiency. This result may be limited by factors such as small sample sizes and inconsistent fatigue intervention measures. In future research, sample size should be expanded and the potential impact of factors (such as age, gender, and athlete level) on fatigue recovery time and technical performance maintenance should be explored in depth.

Although the specific mechanism of physical fatigue affecting shooting is not yet clear, from the bio-mechanical perspective, the shooting motion follows a “proximal-to-distal” sequence involving coordinated movements of multiple joints and muscles, which is transmitted through the kinetic chain of the upper and lower limbs to maintain optimal shooting posture. Fatigue may affect shooting accuracy by altering certain kinematic characteristics, such as angular velocity and muscle coordination. As has been evidenced by Okazaki et al. (2015), after fatigue interventions, athletes mobilize more lower limb muscles to maintain performance, compensating for the reduced upper limb strength by passively controlling the angular velocity of lower limb joints to attempt to stabilize the shooting action. While this compensatory mechanism may help alleviate the impact of fatigue on shooting stability, it could also potentially increase fatigue state, thereby negatively affecting shooting performance. From the perspective of motor control theory, long-range shooting requires athletes to precisely adjust muscle coordination to accommodate the increased shooting distance (Fan et al., 2024). Fatigue may impair the precision of neuromuscular control, disrupting the coordinated transfer of force from the lower limbs to the upper limbs (Cao et al., 2021). As fatigue sets in, athletes struggle to maintain the muscle coordination patterns typical of a non-fatigued state, leading to a reduction in shooting accuracy. Future studies should explore the impact of fatigue on muscle coordination during shooting in greater depth.

### 4.2 The impact of mental fatigue on shooting accuracy

Mental fatigue, also known as cognitive fatigue, is a psychological state induced by prolonged engagement in high-demand cognitive tasks. Mental fatigue is primarily manifested as a decline in cognitive functions (such as attention and memory) and executive functions involved in working memory, decision-making, and multitasking (Daub et al., 2024; Smith et al., 2019; Tseng et al., 2021; Van Cutsem et al., 2017). These authors suggested that attention is crucial as it involves the allocation of cognitive resources to either internal or external stimuli (Furley and Wood, 2016). Moreover, the close relationship between work memory, attention control, and athletic performance has been explored. The dual-processing theory addresses the function of automatic processing (Type 1) and controlled processing (Type 2) in motor performance (Evans, 2003; Evans, 2008). Working memory capacity (WMC) is a critical variable that can predict individual differences in controlling attention in a goal-directed manner and avoiding distractions. Athletes with a high WMC excel at maintaining optimal performance in situations requiring attention control (Furley and Wood, 2016).

In this study, mental fatigue interventions lasting 20–40 min were conducted, and the Visual Analogue Scale (VAS) and the Stroop task were used to induce moderate mental fatigue (VAS score of 3–6). As has been evidenced by Filipas et al. (2021), there is a slight decrease (by 5%) in the shooting accuracy of free throw under mental fatigue. They have suggested that mental fatigue might lead to attention dispersion, accompanied by increased difficulty in maintaining attention and a reduced capacity to ignore irrelevant information. Kurniawan et al. (2011) found that fatigue impairs decision-making by reducing the release of dopamine, which affects the brain’s reward and effort systems. These factors can result in shooting errors by affecting dopamine transmission in cognitive control-related brain areas. Similarly, Englert et al. (2015) have confirmed that states of self-depletion and distractibility significantly lower basketball players’ free-throw accuracy. They confirm the importance of self-control in a high-intensity environment and address the function of self-control in maintaining attention and executing perceptual-motor tasks.

Shaabani et al. (2020) have reported the negative effects of athletes’ reduced ability to regulate attention, control emotions, and allocate cognitive resources on shooting accuracy and stability. Similarly, Bahrami et al. (2020) have observed a significant decrease in three-point shooting accuracy under mental fatigue. They attribute this result to the temporary depletion of cognitive abilities induced by mental fatigue, along with the affected attention maintenance, information processing, executive functions, and perceptual and emotional states. This fatigue weakens the athletes’ decision-making capabilities, technical execution, and tactical judgments. Therefore, to optimize performance during shooting, the authors suggested that mentally taxing activities should be avoided as much as possible before the game. Notably, although the heterogeneity of the four studies assessing the impact of mental fatigue on free throw performance was low (I^2^ ≈ 41%, p > 0.1) in the present review, the limited number and varying quality of the studies suggest that other potential discrepancies cannot be ruled out. Therefore, a thorough assessment is recommended for future research.

## 5 General remarks and limitations

In conclusion, the level and type of fatigue can significantly influence shooting accuracy. Specifically, moderate physical and mental fatigue have a relatively minor impact on shooting accuracy. Severe physical fatigue can cause a notable decline in shooting accuracy, particularly three-point shooting accuracy, with adult athletes being more affected relative to adolescent athletes. Nevertheless, the results of this study depend on the available information of the included studies, and the reliability of these findings may be affected. Future research should expand the sample size and explore the shooting performance under fatigue across different genders and athletic skill levels to establish more precise guidelines for training and coaching.

Some limitations of the present study should be acknowledged:

1) The literature included for the present study was retrieved from the SCI Core Database. However, there’s publication bias in some studies. Although the trim-and-fill test indicated a limited impact of this bias on effect size, conclusions should still be interpreted with caution.
2) While some studies indicate that shooting accuracy differs by gender under various fatigue conditions, there is a lack of research investigating the impact of different levels of mental fatigue on shooting performance in female basketball players. Therefore, subsequent studies are expected to explore gender differences in shooting accuracy under different fatigues.
3) Most of the included studies were conducted under highly-controlled experimental conditions. While such conditions are important for obtaining high-quality data, it is equally important to conduct research in ecologically valid settings to enhance the applicability of findings to real-world scenarios (Pernigoni et al., 2024b). Moreover, the impact of player position and heart rate variability in elite adult players on three-point shooting accuracy was not considered, representing an important direction for future research.

## 6 Conclusion

The findings of this study indicate a significant association between shooting accuracy and different levels and types of fatigue. The shooting accuracy is significantly declined under severe physical fatigue, while mildly affected under moderate physical fatigue. Severe physical fatigue has a greater negative impact on three-point shooting accuracy than on two-point shooting,where accuracy may vary under specific conditions. Moreover, moderate mental fatigue can significantly reduce free-throw accuracy. Shooting accuracy decisively influences basketball game outcomes, and both physical and mental fatigue significantly impair the execution of this skill. In future research, athletes’ fatigue states during training and competition should be thoroughly assessed to enable coaches to develop training plans and adjust game rotation strategies based on scientific data. When adjusting game strategies, coaches should consider increasing rotation depth by reasonably distributing playing time among perimeter players to maintain shooting performance throughout the game. Similarly, optimizing the selection of long-range shots in the final stages of games could prove beneficial. Coaches may consider integrating game-simulated shooting drills into training sessions to enhance athletes’ ability to maintain shooting stability under fatigue during high-intensity games. Furthermore, future research should focus on the impact of mental fatigue on athletic performance and explore effective strategies to mitigate its adverse effects. It is recommended that training interventions focusing on dual fatigue should prioritize enhancing athletes’ mental endurance and cognitive function and achieved by incorporating psychological recovery strategies to prevent and mitigate mental fatigue.

## Data Availability

The original contributions presented in the study are included in the article/Supplementary Material, further inquiries can be directed to the corresponding author..
