# Supplementary material for: Changes in shooting accuracy among basketball players under fatigue: a systematic review and meta-analysis
Source: Front Physiol. 2025 Feb 26;16:1435810. doi: 10.3389/fphys.2025.1435810 (PMC11897034; doi:10.3389/fphys.2025.1435810)
Supplement: Supplementary file 1 [file Table1.docx]

A Systematic Review and Meta-Analysis of Changes in Shooting Accuracy Among Basketball Players Under Fatigue

**Supplementary file 1.Search strategy**

| *Search the database* | *Search Strategy* |
| --- | --- |
| *PUBMED* | *("Fatigue levels"[Title/Abstract] OR "Physical fatigue"[Title/Abstract] OR "Mental fatigue"[Title/Abstract]) AND ("Basketball players"[Title/Abstract] OR "Basketball"[Title/Abstract]) AND ("Shooting accuracy"[Title/Abstract] OR "Shooting hit rate"[Title/Abstract] OR "Shooting performance"[Title/Abstract]) AND ("Impact"[Title/Abstract] OR "Effect"[Title/Abstract])* |
| *EBSCO* | *(TI (("Fatigue levels" OR "Physical fatigue" OR "Mental fatigue") AND ("Basketball players" OR "Basketball") AND ("Shooting accuracy" OR "Shooting hit rate" OR "Shooting performance") AND ("Impact" OR "Effect")) AND (("Fatigue levels" OR "Physical fatigue" OR "Mental fatigue") AND ("Basketball players" OR "Basketball") AND ("Shooting accuracy" OR "Shooting hit rate" OR "Shooting performance") AND ("Impact" OR "Effect") ))* |
| *WOS* | *TS= ((Fatigue levels OR Physical fatigue OR Mental fatigue) AND (Basketball players OR Basketball) AND (Shooting accuracy OR shooting hit rate OR Shooting performance) AND (Impact OR Effect))* |

**Supplementary file 2.PICOS framework**

| *PICOS* | *Definition* | *Description* |
| --- | --- | --- |
| *P - Population* | *Professional Male Basketball Players* | *Includes professional male basketball players of all ages participating in national or international professional leagues.* |
| *I - Interventions* | *Fatigue induction (physical or mental)* | *Independently induced physical fatigue (e.g., prolonged physical training) or mental fatigue (e.g., high-intensity cognitive tasks) using scientifically validated methods. Exclude studies that induced both physical and mental fatigue.* |
| *C - Comparison* | *Fatigued vs. non-fatigued state* | *Comparing shooting performance in fatigued and non-fatigued states, or before and after fatigue* |
| *O - Outcomes* | *Shooting Hit Rate* | *Focuses on measuring changes in shooting hits, including detailed statistical analysis of the results.* |
| *S - Study design* | *Randomized controlled trials and prospective cohort studies* | *Include studies with designs that provide evidence of causality, such as randomized controlled trials and prospective cohort studies. Exclude all cross-sectional studies.* |

# Supplementary file 3. Exclusion criteria as list

| **Included conditions** | **Excluded conditions** |
| --- | --- |
| The study population consists of basketball players in healthy condition | Participant including wheelchair basketball or injury rehabilitation and other special groups |
| Interventions include physical or mental fatigue alone | Rotator cuff rupture, acromioclavicular joint subluxation, shoulder dislocation, instability, labral tear (partial or complete) |
| Outcome measures utilize the ratio of goals scored to total attempts, with the primary outcome being the shooting accuracy | Interventions that include elements other than physical or mental fatigue, such as strength training, nutritional supplements, or pharmacological treatment |
| Double-arm randomized controlled trials and single-arm pre-post design are both included in this study | Utilizing basketball robots or artificial intelligence for analyzing basketball games |
|  | Utilizing basketball robots or artificial intelligence for analyzing basketball games |
|  | Qualitative research, case reports, review articles, non-intervention studies, and conference papers will be excluded |
|  | Intervention both physical and mental fatigue |
